# High Frequency of Imprinted Methylation Errors in Human Preimplantation Embryos

**DOI:** 10.1038/srep17311

**Published:** 2015-12-02

**Authors:** Carlee R. White, Michelle M. Denomme, Francis R. Tekpetey, Valter Feyles, Stephen G. A. Power, Mellissa R. W. Mann

**Affiliations:** 1 Department of Obstetrics & Gynecology, University of Western Ontario, Schulich School of Medicine and Dentistry, London, Ontario, Canada; 2 Department of Biochemistry, University of Western Ontario, Schulich School of Medicine and Dentistry, London, Ontario, Canada; 3 Children’s Health Research Institute, London, Ontario, Canada; 4 The Fertility Clinic, London Health Sciences Centre, London, Ontario, Canada

## Abstract

Assisted reproductive technologies (ARTs) represent the best chance for infertile couples to conceive, although increased risks for morbidities exist, including imprinting disorders. This increased risk could arise from ARTs disrupting genomic imprints during gametogenesis or preimplantation. The few studies examining ART effects on genomic imprinting primarily assessed poor quality human embryos. Here, we examined day 3 and blastocyst stage, good to high quality, donated human embryos for imprinted *SNRPN*, *KCNQ1OT1* and *H19* methylation. Seventy-six percent day 3 embryos and 50% blastocysts exhibited perturbed imprinted methylation, demonstrating that extended culture did not pose greater risk for imprinting errors than short culture. Comparison of embryos with normal and abnormal methylation didn’t reveal any confounding factors. Notably, two embryos from male factor infertility patients using donor sperm harboured aberrant methylation, suggesting errors in these embryos cannot be explained by infertility alone. Overall, these results indicate that ART human preimplantation embryos possess a high frequency of imprinted methylation errors.

Alarming figures indicate that an estimated 48.5 million couples worldwide are unable to conceive after 5 years of unprotected sex^1^. For these couples, medically assisted reproductive technologies (ARTs) represent the best chance to conceive. However, when treatment is successful (<40%), there is an increased risk of perinatal complications even within singletons, including preterm birth, intrauterine growth restriction, low birth weight^1^,^2^,^3^ and the genomic imprinting disorders Beckwith-Wiedemann Syndrome (BWS)^4^,^5^,^6^,^7^,^8^, Angelman Syndrome (AS)^6^,^8^,^9^,^10^,^11^, and Silver-Russell Syndrome (SRS)^12^,^13^,^14^,^15^,^16^,^17^,^18^.

Genomic imprinting is an epigenetic phenomenon that restricts expression to one parental allele while the other allele is in an inactivated state. Imprinted genes are regulated by a master control switch known as a gametic differentially methylated region (gDMR) or imprinting control region (ICR). Importantly, abnormal cytosine methylation levels at the ICR can lead to imprinting disorders such as BWS, AS and SRS.

Risk association studies have found increased risks of imprinting disorders in ART children. The risk of BWS is 3–16 times greater in children in the ART population compared to those in the general population^4^,^5^,^7^,^15^,^19^,^20^,^21^,^22^,^23^,^24^. Epigenetic errors at *KCNQ1OT1*, namely maternal hypomethylation, are observed in more than 90% of ART BWS cases compared to 50% in the general population^4^,^5^,^7^,^15^,^19^,^20^,^21^,^22^,^23^,^25^,^26^, while *H19* maternal hypermethylation occurs in 17% of ART BWS cases compared to 5% in the general population^4^,^21^,^25^,^27^. Of the small number of patients analyzed, 46% of AS patients conceived by ARTs possessed imprinting defects at the *SNRPN* ICR compared to 5% in the general population^9^,^10^,^11^,^28^, while 92% of SRS patients conceived by ARTs harboured *H19* hypomethylation compared to 40% in the general population^12^,^13^,^14^,^15^,^16^,^17^,^18^, The overall risk for an imprinting disorder such as BWS, AS or SRS in ART children is estimated to be around 1 in 5,000^3^. Thus, disparity has arisen concerning the frequency of imprinting errors produced by ARTs in humans compared to mice, as mouse studies have identified between 10% to 90% of treated preimplantation embryos showing abnormal imprint maintenance^29^,^30^,^31^,^32^,^33^. However, one key difference in studies between these species is the time of analysis. The majority of mouse studies have focused on preimplantation or mid-gestation development, while human studies are primarily retrospective studies of ART children with imprinting disorders. Consequently, we sought to determine whether donated human ART-produced preimplantation embryos harbour aberrant imprinted methylation at similar incidences to that observed in the mouse^29^,^30^,^31^,^32^,^33^. Additionally, we analyzed whether short or extended culture produces a greater frequency of imprinted methylation errors, and whether aberrant imprinted methylation correlates with parental biometrics or clinical treatment. We analyzed methylation levels at *SNRPN*, *KCNQ1OT1* and *H19* ICRs in individual good to high quality day 3 cleavage and blastocyst stage ART-produced human embryos.

## Results

### Imprinted methylation in control samples

Imprinted DNA methylation at the *SNRPN*, *KCNQ1OT1* and *H19* ICR was first assessed in untreated human buccal cell (Bu) samples from 4 young, non-patient adults. Bisulfite clonal sequencing was used to analyze 20–24 CpGs per gene. For all controls, a total of 30–65 clones were sequenced to obtain representative DNA strands. Sequences with identical CpG methylation profiles and unconverted cytosines were considered to be identical and were included once to eliminate clonal bias. Each region of analysis included a single nucleotide polymorphism(s) (SNP) that when present in heterozygous samples could distinguish between parental alleles (Supplementary Table 1 online). Since we did not have access to patient samples, we consider the methylated strands as the presumptive paternal *H19*, maternal *SNRPN* and maternal *KCNQ1OT1* alleles, and the unmethylated strands as the maternal *H19*, paternal *SNRPN* and paternal *KCNQ1OT1* alleles, as was done in previous studies^34^,^35^.

**Figure 1:**
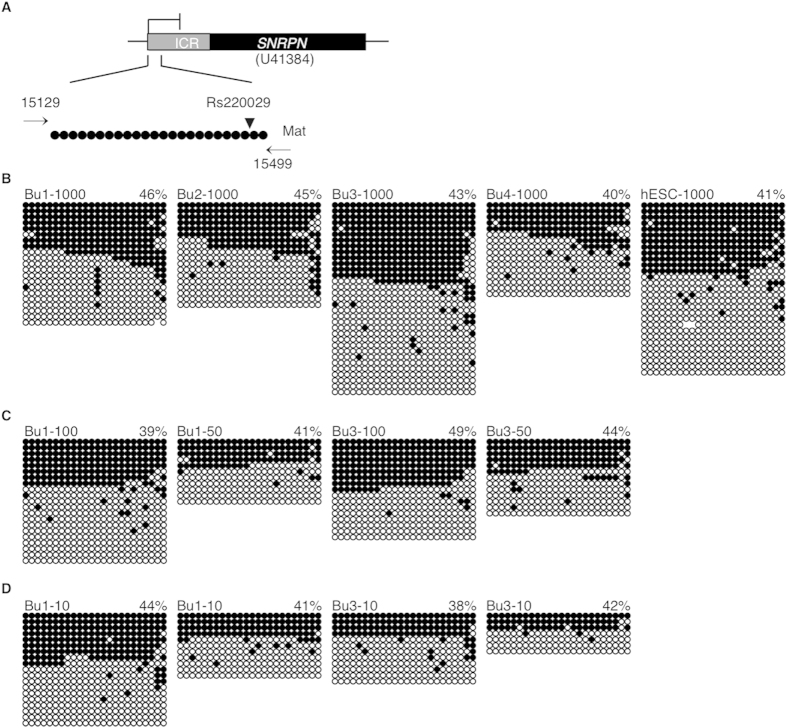
*SNRPN* imprinted methylation in buccal cell and human embryonic stem cell (hESC) control samples. (**A**) Map of the *SNRPN* region analyzed. Accession numbers are located below genes, primer locations are marked with arrows, and SNPs are indicated by arrowheads. Methylation analyses in (**B**) four buccal cell (Bu1-4) and human embryonic stem cell (hESC) control samples with ~1000 cells, (**C**) in buccal cell samples with ~100 and ~50 cells, representing blastocysts, and (**D**) with buccal cell samples ~10 cells, representing day 3 cleavage embryos. Each group of circles represents an individual human sample. Each line is an individual DNA strand. Methylated CpGs are filled black circles and unmethylated CpGs are open circles. Percent methylation is indicated above each set of DNA strands for a gene or parental allele and was calculated as the number of methylated CpGs divided by the total number of CpG dinucleotides.

For the *SNRPN* ICR, a 360 bp-region was analyzed comprising 24 CpGs and a G/A SNP (Rs220029) that occurs at a general population frequency of 84.8% and 15.2%, respectively (Fig. 1A). All control samples were homozygous at this SNP (Supplementary Table 1 online), and thus no allelic assignment could be made. Total *SNRPN* methylation levels in buccal cell controls (~1000 cells) were Bu1-1000 46%, Bu2-1000 45%, Bu3-1000 43% and Bu4-1000 40% (Fig. 1B). Since buccal samples exhibited a mean *SNRPN* methylation level less than anticipated (43%), we analyzed *SNRPN* methylation in human embryonic stem cells (hESCs), an undifferentiated cell type that more closely matched preimplantation embryos. In hESCs, *SNRPN* methylation levels were 41% (Fig. 1B), consistent with those in buccal cells. To assess cell numbers similar to blastocyst and day 3 embryos, methylation levels were analyzed in approximately 100, 50 (Fig. 1C) and 5–10 cells (denoted hereafter as 10 cells) (Fig. 1D) for Bu1 and Bu3 samples. Total *SNRPN* methylation levels were Bu1-100 39%, Bu1-50 41% (Fig. 1C), Bu1-10 44% and 41% (Fig. 1D), and Bu3-100 49%, Bu3-50 44% (Fig. 1C), Bu3-10 38% and 42% (Fig. 1D). Thus within sample, methylation level mean and standard deviation were 42.2 ± 2.8 for Bu1 and 43.2 ± 4.0 for Bu3.

**Figure 2:**
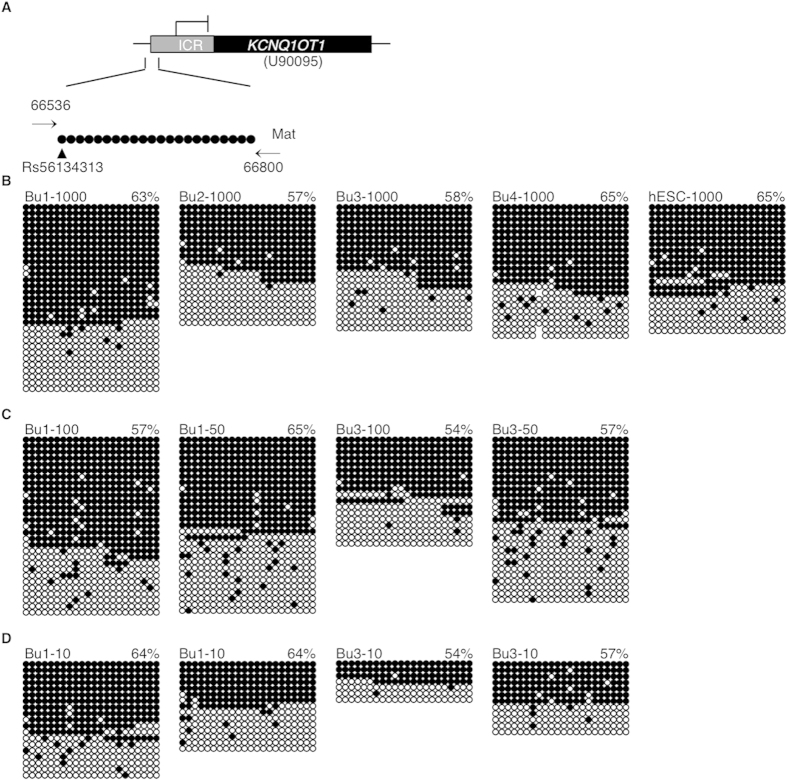
*KCNQ1OT1* imprinted methylation in buccal cell and hESC control samples. (**A**) Map of the *KCNQ1OT1* region analyzed. Methylation analyses in (**B**) buccal cell (Bu) and human embryonic stem cell (hESC) control samples with ~1000 cells, (**C**) in buccal cell samples with ~100 and ~50 cells, representing blastocysts, and (**D**) with buccal cell samples ~10 cells, representing day 3 cleavage embryos. See Figure legend 1 for details.

For the *KCNQ1OT1* ICR, a 265 bp-region was analyzed encompassing 22 CpGs^34^ and a G (94.7%)/A (6.3%) SNP (Rs56134313), that eliminated the first CpG (Fig. 2A). All controls were homozygous at the *KCNQ1OT1* SNP (Supplementary Table 1 online). Total *KCNQ1OT1* methylation levels in control samples were Bu1-1000 63%, Bu2-1000 57%, Bu3-1000 58% and Bu4-1000 65% (Fig. 2B). Since the mean *KCNQ1OT1* methylation level was greater than anticipated (60%), *KCNQ1OT1* methylation was assessed in hESCs. *KCNQ1OT1* methylation levels were hESC-1000 65% (Fig. 2B), consistent with those in buccal cells. At cell numbers similar to blastocyst and day 3 embryos, *KCNQ1OT1* methylation levels were Bu1-100 57%, Bu1-50 65%, (Fig. 2C), Bu1-10 64% and 64% (Fig. 2D), and Bu3-100 54%, Bu3-50 57% (Fig. 2C), Bu3-10 54% and 57% (Fig. 2D). Thus within sample, methylation level mean and standard deviation were 62.6 ± 3.2 for Bu1 and 56.0 ± 1.8 for Bu3.

**Figure 3:**
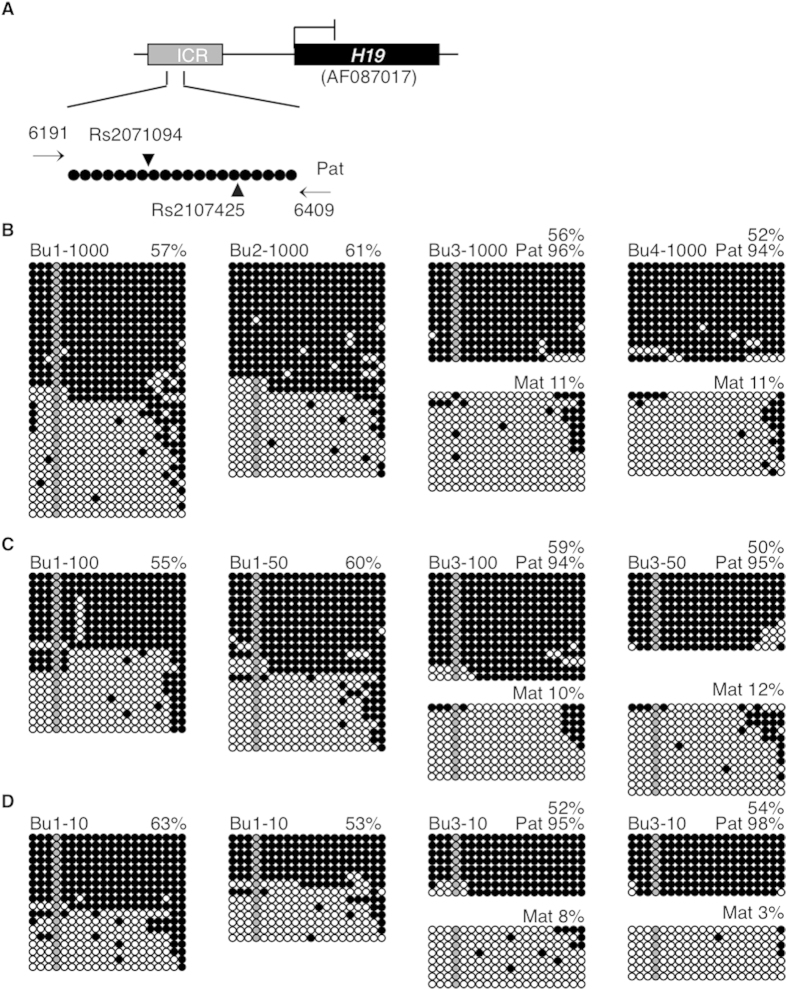
*H19* imprinted methylation in buccal cell control samples. (**A**) Map of the *H19* region analyzed. (Methylation analyses in (**B**) buccal cell (Bu) and human embryonic stem cell (hESC) control samples with ~1000 cells, (**C**) in buccal cell samples with ~100 and ~50 cells, representing blastocysts, and (**D**) with buccal cell samples ~10 cells, representing day 3 cleavage embryos. Grey circles are not included in methylation analyses as they represent a C/T SNP that cannot be distinguished following bisulfite conversion. Alleles are separated into presumptive maternal (Mat) and paternal (Pat) strands in samples with heterozygous SNPs. See Figure legend 1 for details.

Samples assessed for *KCNQ1OT1* methylation levels were also analyzed for DNA methylation at the *H19* ICR. We initially began our analysis for a 234 bp-region within the *H19* imprinting control region that included 18 CpGs^34^ and a common A (33.6%)/C (66.4%) SNP (Rs2071094) (Fig. 3A). However, we observed biased allelic recovery and subsequently found two additional SNPs present in the forward and reverse inner nested primers. Thus, we designed new internal primers for a larger 249 bp-region within the *H19* ICR containing 20 CpGs, the Rs2071094 (A, 33.6%; C, 66.4%) and the Rs2107425 SNP (G, 55.5%; A, 44.5%) SNP (Fig. 3A). For buccal cell samples, Bu3 was heterozygous at both *H19* SNPs, Bu4 was heterozygous at one SNP, while Bu1 and Bu2 were homozygous for both *H19* SNPs (Supplementary Table 1 online). Samples Bu1-1000 and Bu2-1000 had total *H19* methylation levels of 57% and 61%, respectively. Sample Bu3-1000 had 96% methylation on the presumptive paternal *H19* allele and 11% methylation on the presumptive maternal *H19* allele (56% total methylation), while Bu4-1000 had 94% and 11% methylation on the presumptive paternal and maternal *H19* alleles, respectively (52% total methylation) (Fig. 3B). Thus, total methylation levels fell within a mean (56%) expected for paternally methylated and maternally unmethylated alleles. For smaller cell numbers, total *H19* methylation levels were Bu1-100 55%, Bu1-50 60% (Fig. 3C), Bu1-10 63% and 53% (Fig. 3D), and Bu3-100 59%, Bu3-50 50% (Fig. 3C), Bu3-10 52% and 54% (Fig. 3D), with 94–98% and 3–12% methylation on the presumptive paternal and maternal *H19* alleles, respectively. Thus within sample, methylation level mean and standard deviation were 57.6 ± 4.0 for Bu1 and 54.2 ± 3.5 (Pat 95.6 ± 1.5; Mat 8.8 ± 3.6) for Bu3.

Given the *SNRPN*, *KCNQ1OT1* and *H19* methylation levels in all control samples, conservatively, we considered a methylation range of 4 times the standard deviations above/below the mean as a normal methylation level. For *SNRPN*, the mean methylation level was 42.2% ± 3.0, generating a 30%-54% normal methylation range. For *KCNQ1OT1*, the mean methylation level was 60.0% ± 4.4, giving a normal methylation range of 42%–78%. The mean methylation level for *H19* was 56.0% ± 4.1, generating a 40%–72% normal methylation range. For embryos with heterozygous SNPs, conservatively ≥70% methylation on the presumptive maternal *SNRPN*, maternal *KCNQ1OT1* and paternal *H19* alleles and ≤20% methylation on the presumptive paternal *SNRPN*, paternal *KCNQ1OT1* and maternal *H19* alleles were considered as normal methylation levels.

### Aberrant imprinted methylation in day 3 embryos

During fertility treatment, embryos were cultured to day 3, after which embryos were transferred to the mother, cryopreserved and stored for future cycles, or cultured to the blastocyst stage then cryopreserved and stored for future cycles. For identification purposes, embryos were given an alphanumeric ID that included patient number (1–23), freeze stage [day 3 cleavage (C) or blastocyst (B)], and embryo number (1–6), for example “9C2” represents patient 9, day 3 cleavage embryo 2. Individual, cryopreserved day 3 cleavage embryos were analyzed for maintenance of imprinted methylation. For all day 3 and blastocyst-stage embryos, a total of 30–65 clones were sequenced to obtain representative DNA strands and to sequence all possible unique DNA strands following thawing and bisulfite treatment. Data were obtained for 9 out of 12 day 3 embryos for *SNRPN*; 7 out of 12 day 3 embryos for *KCNQ1OT1*; and 7 out of 12 day 3 embryos for *H19*.

**Figure 4:**
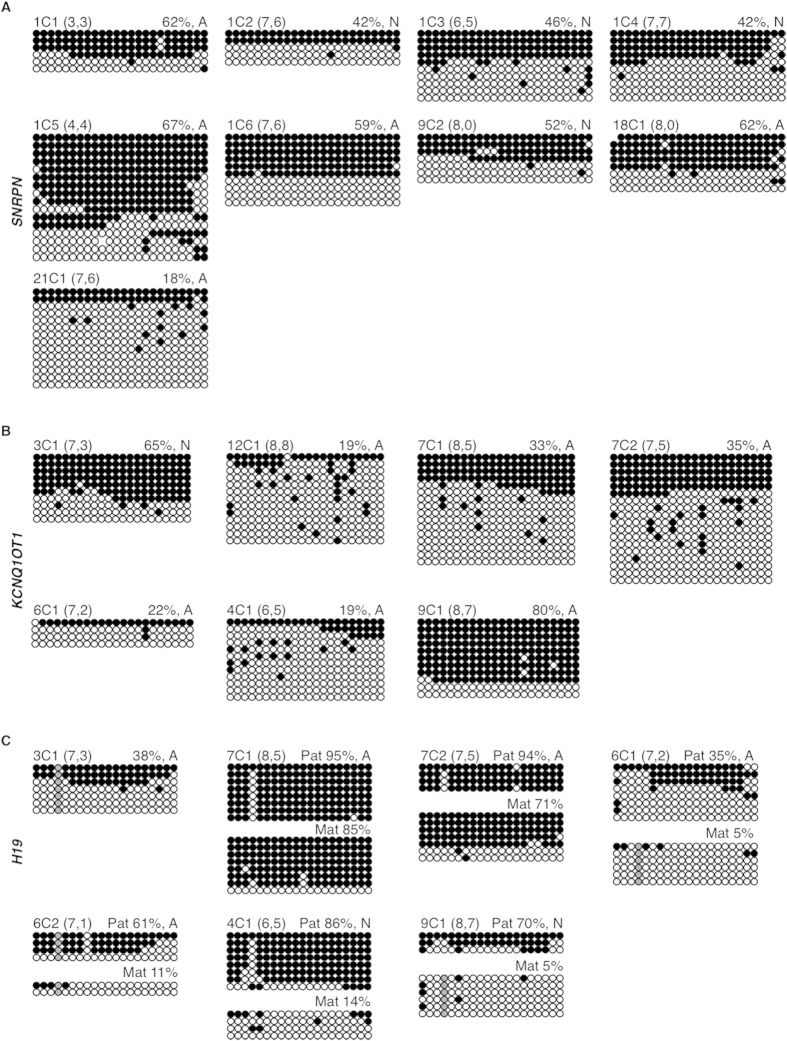
Methylation of the (**A**) *SNRPN*, (**B**) *KCNQ1OT1* and (**C**) *H19* ICRs in day 3 human cleavage-stage embryos. Each group of DNA strands is an individual day 3 embryo with embryo ID (top left), and percent methylation and presumptive maternal/paternal allele designation (top right) indicated. Normal (N) and abnormal (A) embryos are designated after percent methylation (top right). The pre-freeze and post-thaw cell numbers, respectively, are indicated in brackets beside each embryo name. See Figure legend 1 for details.

*SNRPN* is normally methylated on the silent maternal allele, while the paternal allele is unmethylated. All day 3 embryos were homozygous at the Rs220029 SNP (Supplementary Table 1 online) and thus were examined for total methylation levels. Of the 9 day 3 cleavage embryos analyzed, normal methylation levels were observed for 4 embryos (Fig. 4A). By comparison, 5 embryos had abnormal *SNRPN* methylation levels, with 4 embryos exhibiting aberrant hypermethylation (1C1, 62%; 1C5, 67%; 1C6, 59%; 18C1, 62%) and 1 embryo (21C1) displaying aberrant hypomethylation of 18%. Overall, 56% of day 3 cleavage embryos had abnormal *SNRPN* imprinted methylation.

*KCNQ1OT1* is also normally methylated on the silent maternal allele, while the paternally inherited allele is unmethylated. All 7 day 3 embryos were homozygous at the Rs56134313 SNP (Supplementary Table 1 online), and thus, total methylation levels were analyzed. One embryo had methylation levels within the normal range (Fig. 4B). Of the remaining 6 embryos, 1 embryo had abnormal hypermethylation (9C1, 80%) while 5 embryos exhibited aberrant *KCNQ1OT1* hypomethylation (12C1, 19%; 7C1, 33%; 7C2, 35%; 6C1, 22%; 4C1, 19%). In total, 86% of day 3 cleavage embryos had aberrant *KCNQ1OT1* imprinted methylation.

*H19* is normally methylated on the paternal allele, while the maternally inherited allele is unmethylated. Three day 3 cleavage embryos (4C1, 7C1, 7C2) were heterozygous at both Rs2071094 and Rs2107425, 1 embryo (6C2) was heterozygous at Rs2071094 and 2 embryos (6C1, 9C1) were heterozygous at Rs2107425 (Supplementary Table 1 online), allowing for allelic assignment. Only one embryo (3C1) was homozygous at the Rs2071094 and Rs2107425 SNPs and was examined for total methylation levels. Out of 7 day 3 embryos, 2 had a normal methylation pattern with methylation ≥70% on the presumptive paternal allele and hypomethylation on the presumptive maternal allele (Fig. 4C). Of the remaining 5 embryos, 2 showed loss of methylation on the presumptive paternal *H19* allele (6C1, 35%; 6C2, 61%) and 2 displayed a gain of methylation on the presumptive maternal allele (7C1, 85%; 7C2, 71%). Finally, for the homozygous embryo (3C1), there was a loss of total *H19* methylation (38%). Overall, 71% of day 3 cleavage embryos were abnormally hypo- and/or hypermethylated at *H19*. Furthermore, of the 6 embryos successfully assessed for both *KCNQ1OT1* and *H19* methylation, 3 embryos (50%) displayed aberrant methylation levels at both genes (7C1; 7C2; 6C1).

### Abnormal imprinted methylation in blastocyst stage embryos

**Figure 5:**
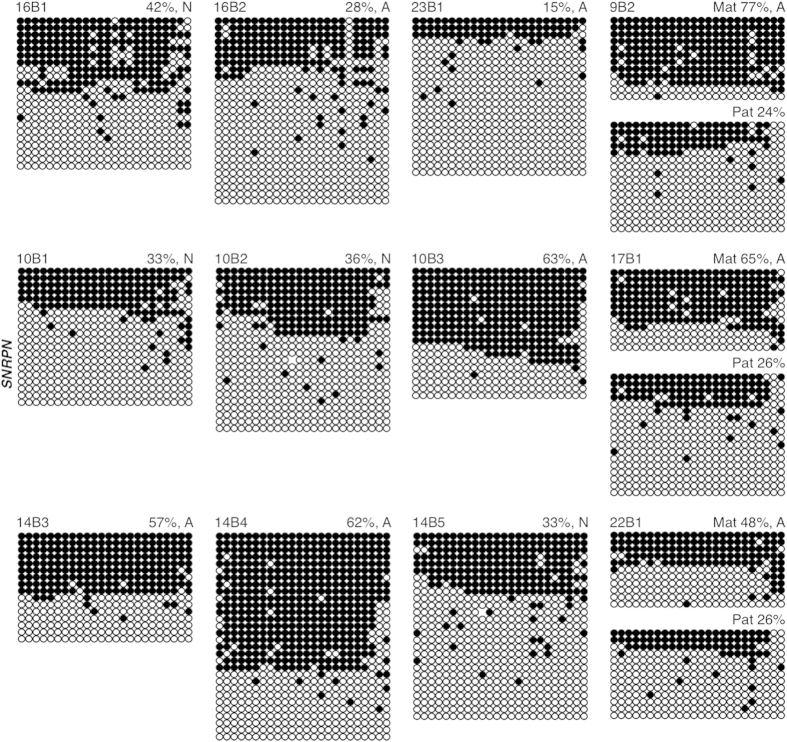
Methylation of the *SNRPN* ICR in human blastocyst-stage embryos. Each group of DNA strands is an individual blastocyst with embryo ID (top left), and percent methylation and presumptive maternal/paternal allele designation (top right) indicated. Normal (N) and abnormal (A) embryos are designated after percent methylation (top right). See Figure legend 1 for details.

Individual, cryopreserved blastocysts were also analyzed for maintenance of imprinted methylation. Data were obtained for 12 out of 15 blastocysts for *SNRPN*; 13 out of 14 blastocysts for *KCNQ1OT1*; and 14 out of 14 blastocysts for *H19*. For *SNRPN*, 3 blastocyst-stage embryos (22B1, 9B2, 17B1) were heterozygous at Rs220029, while the remaining 9 embryos were homozygous at the Rs220029 SNP (Supplementary Table 1 online). Four embryos had total methylation levels within the normal range (30%–54%) (Fig. 5). Of the 8 remaining embryos, 3 homozygous embryos showed a gain of total *SNRPN* methylation (10B3, 63%; 14B3, 57%; and 14B4, 62%), and 2 homozygous blastocysts exhibited *SNRPN* hypomethylation (16B2, 28%; and 23B1, 15%), while 1 heterozygous blastocyst (9B2) exhibited a gain of paternal *SNRPN* methylation (24% Pat) and 2 heterozygous blastocysts possessed both a loss of maternal *SNRPN* methylation and a gain of paternal *SNRPN* methylation (17B1, 65% Mat, 26% Pat; and 22B1, 48% Mat, 26% Pat) (Fig. 3). In total, 67% of blastocyst embryos exhibited abnormal *SNRPN* imprinted methylation.

**Figure 6:**
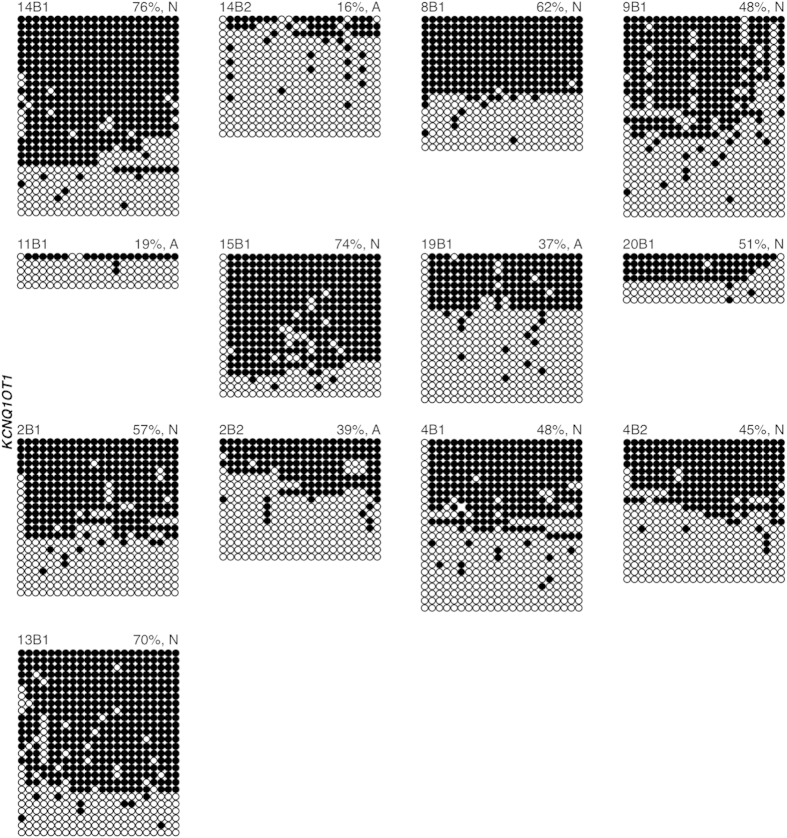
Methylation of the *KCNQ1OT1* ICR in human blastocyst-stage embryos. Each group of DNA strands is an individual blastocyst with embryo ID (top left), and percent methylation and presumptive maternal/paternal allele designation (top right) indicated. Normal (N) and abnormal (A) embryos are designated after percent methylation (top right). See Figure legend 1 for details.

For *KCNQ1OT1*, all embryos were homozygous at the Rs56134313 SNP (Supplementary Table 1 online), allowing total methylation levels to be determined. Normal *KCNQ1OT1* methylation levels (42%–78%) were observed in 9 blastocysts (Fig. 6). For the remaining 4 blastocysts, a loss of *KCNQ1OT1* methylation was observed (14B2, 16%; 11B1, 19%; 19B1, 37%; and 2B2, 39%). Overall, 4 out of 13 (31%) blastocysts had abnormal *KCNQ1OT1* methylation levels.

**Figure 7:**
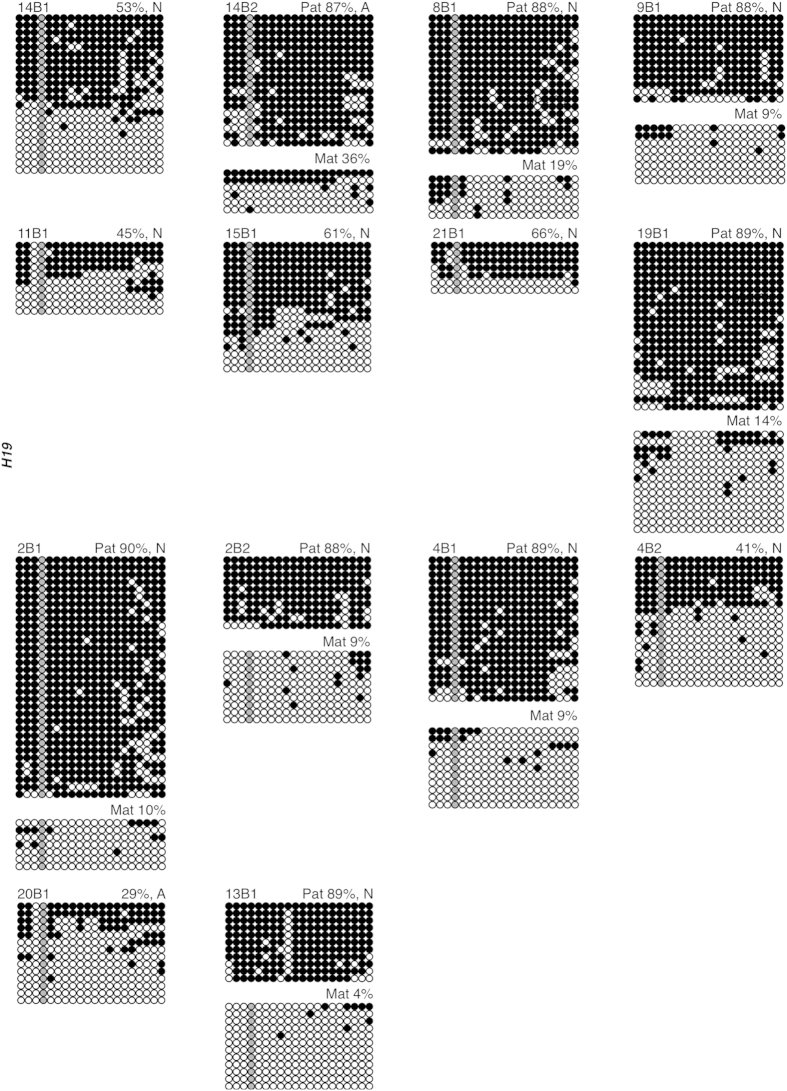
Methylation of the *H19* ICR in human blastocyst-stage embryos. Each group of DNA strands is an individual blastocyst with embryo ID (top left), and percent methylation and presumptive maternal/paternal allele designation (top right) indicated. Normal (N) and abnormal (A) embryos are designated after percent methylation (top right). See Figure legend 1 for details.

The same 14 embryos analyzed for *KCNQ1OT1* imprinted methylation were assessed for *H19* imprinted methylation. Three blastocysts (4B1, 8B1, 14B2) were heterozygous at Rs2071094 and Rs2107425, 3 blastocysts (9B1, 19B1, 2B1) were heterozygous at Rs2071094, and 2 blastocysts (2B2 and 13B1) were heterozygous for Rs2107425 (Supplementary Table 1 online). The remaining 6 blastocysts (14B1, 11B1, 15B1, 21B1, 4B2 and 20B1) were homozygous for both *H19* SNPs (Supplementary Table 1 online). All blastocysts, except 2, fell within the normal *H19* methylation range (40%–72%) (Fig. 7). One blastocyst displayed a loss of total *H19* methylation (20B1, 29%) and one displayed abnormal gain of maternal *H19* methylation (14B2, 87% Pat, 36% Mat). Overall, 14% of blastocysts had an abnormal *H19* methylation profile. Blastocyst 20B1, with aberrant *H19* methylation, had normal *KCNQ1OT1* methylation, while blastocyst 14B2 had abnormal methylation at both *H19* and *KCNQ1OT1*. In total for all three genes, 76% day 3 embryos and 50% blastocysts exhibited abnormal imprinted methylation (Supplemental Figure 1 online).

### Intra-patient comparison of imprinted methylation in embryos at different preimplantation stages

The design of this study allowed multiple embryos from the same patient to be compared for their imprinted methylation status. Out of 22 patients for whom data were obtained, 10 patients had more than one embryo analyzed (Supplementary Table 2 online). For two patients, 6 and 7, all *in vitro*-produced embryos experienced perturbations in imprinted methylation (*KCNQ1OT1*/*H19* or *H19*). The remaining 8 patients had a portion of embryos with normal and a portion of embryos with abnormal methylation levels. For patient 1, 3/6 day 3 embryos had aberrant *SNRPN* imprinted methylation. For patients 2, 10, 14 and 16, 1/2 (abnormal *KCNQ1OT1*), 1/3 (abnormal *SNRPN*), 3/5 (aberrant *KCNQ1OT1*/aberrant *H19*; aberrant *SNRPN*) and 1/2 (abnormal *SNRPN*) blastocysts had aberrant methylation levels, respectively. Finally, three patients had both day 3 cleavage and blastocyst-stage embryos. For patient 21 and 4, the day 3 embryos had aberrant methylation (abnormal *SNRPN*; abnormal *KCNQ1OT1*), while the blastocysts displayed normal methylation levels. Finally, for patient 9, 1 day 3 embryo and 1 blastocyst possessed normal methylation levels, while 1 day 3 embryo and 1 blastocyst had perturbed methylation (abnormal *KCNQ1OT1*; abnormal *SNRPN*). Overall, all 10 patients had at least one embryo with aberrant imprinted methylation. Since there were embryos with and without imprinted methylation errors from the same patient, and there were genes with and without aberrant imprinted methylation in the same embryo, methylation errors were likely stochastic in nature. Furthermore, the presence of methylation errors in both day 3 cleavage and blastocyst-stage embryos indicates that methylation errors arise as early as the 6–8 cell stage, and that extended culture does not pose a greater risk for imprinting errors than short culture.

### Correlation between parental biometrics, clinical treatment and aberrant imprinted methylation

Medical records were examined for parental biometrics, clinical treatment and pregnancy outcomes. Clinical pregnancy rates for fresh embryo transfers as determined by gestational sac by ultrasound were 65% for the same cycle in which the surplus embryos were cryopreserved and donated. Live birth rate was 61% and live births/embryo transfer was 36% (Supplementary Table 3 online). Of all live births, 45% (9/20) of newborns (2 singletons, 3 sets of twins, and 1 of the triplets) were outside clinically normal birth weight, with 1 high birth weight (>4000 g), 5 low birth weight (<2500), 1 very low birth weight (<1500 g) and 2 extremely low birth weight (<1000 g). To discern any confounding factors related to parental biometrics or clinical treatment, embryos with methylation levels in the normal range were compared to embryos with aberrant methylation for maternal age, patient diagnosis, induction method, hormone dose, stimulation response (E_2_ levels), fertilization method (IVF/ICSI), and embryo grade (Supplementary Table 4 online). Note that for all embryos, the same conditions and reagents were used for *in vitro* culture and slow-freezing cryopreservation, and thus no comparison could be made. For this analysis, the premise was that each embryo could have a different response to influences/exposures, although we acknowledge that embryos from the same mother may have similar exposures to maternal factor treatment. To make a comparison at the patient level for maternal age, hormone dose and estrogen response, separate analyses was also done for patients with only one embryo (12/22), since the remaining 10 patients with more than one embryo had a least one embryo with abnormal methylation. Data from both stages were combined for analyses, except for embryo grade.

**Figure 8:**
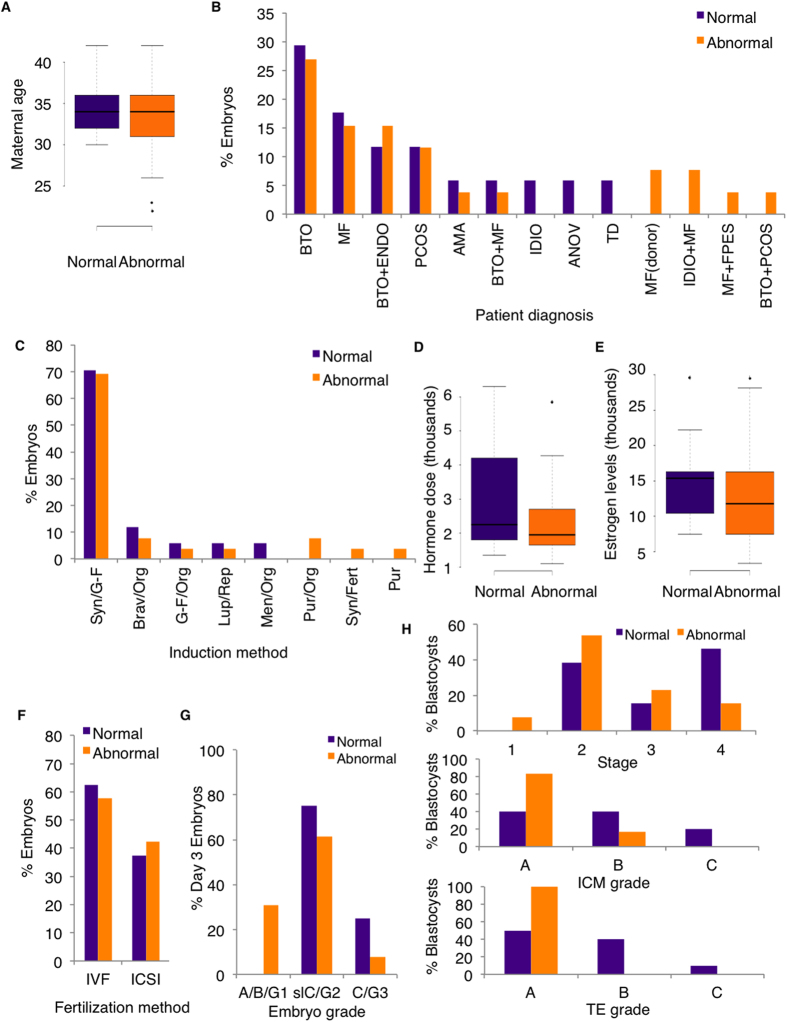
Patient characteristics and embryo outcome for embryos with normal and abnormal imprinted methylation. Day 3 cleavage and blastocyst-stage embryos exhibiting normal imprinted methylation (purple bars; n = 17) were compared to those with abnormal methylation (orange bars; n = 26) for (**A**) maternal age (t-test), (**B**) patient diagnosis (KS test), (**C**) induction method (KS test), (**D**) hormone dose (t-test), (**E**) estrogen levels (t-test), and (**F**) fertilization method (KS test). Means are indicated by black line for maternal age, hormone dose and estrogen levels. (**G**) Day 3 embryos with normal (n = 4) and abnormal methylation (n = 13) (no statistical analysis). (**H**) Blastocysts with normal [n = 13 (stage), n = 10 (grade)] and abnormal methylation [n = 13 (stage), n = 6 (grade)] were compared for embryo stage and grade (KS test). No significant difference was observed for any parameter between embryos with normal and abnormal methylation. BTO, bilateral tubal obstruction/occlusion; MF, male factor; ENDO, endometriosis; PCOS, polycystic ovarian syndrome; AMA, advanced maternal age; IDIO, idiopathic; ANOV, anovulatory; TD, tubal disease; (donor), donor sperm; FPES Fresh/frozen percutaneous epididymal/testicular sperm aspiration sample; Syn, Synarel® (Nafarelin); G-F, Gonal-F® (Follitropin-alpha); Brav, Bravelle® (Urofollitropin); Org, Orgalutran® (Ganirelix Acetate); Lup, Lupron® (Leuprolide Acetate); Rep, Repronex® (Menotropins); Men, Menopur® (Menotropins); Pur, Puregon® (Follitropin-beta); Fert, Fertinorm® (Urofollitrophin); IVF, *in vitro* fertilization; ICSI intracytoplasmic sperm injection. See methods for embryo grades.

Maternal age range for patients in this study was 23–42 years. Mean maternal age for embryos with normal methylation levels was 34 years while that for embryos with aberrant methylation was 33 years (Fig. 8A), which was not statistically different (p = 0.21). Excluding patients with more than one embryo, maternal age for embryos with normal methylation levels was 33 years while that for embryos with abnormal methylation was 30 years (results not shown) (p = 0.38). Multiple etiologies contributing to infertility were diagnosed in patients. The four most common patient diagnoses were bilateral tubal obstruction/occlusion (BTO, 29.4% normal, 26.9% abnormal), male factor (MF, 17.6% normal, 15.4% abnormal), blocked tubes with endometriosis (BTO + ENDO, 11.8% normal, 15.4% abnormal) and polycystic ovarian syndrome (PCOS, 11.8% normal, 11.5% abnormal) (Fig. 8B). Thus, patient diagnoses were not statistically different between embryos with normal and abnormal methylation levels (p > 0.99). For induction method, Nafarelin (Synarel®) and Follitropin-alpha (Gonal-F®) was the most common hormone combination for patients in both normal (70.6%) and abnormal (69.2%) embryo groups, followed by Urofollitropin (Bravelle®) and Ganirelix Acetate (Orgalutran®) (11.8% normal and 7.7% abnormal) (Fig. 8C). Thus, no significant difference was observed for hormone induction method (p = 0.80). Mean hormone dose and estrogen response (E_2_) was calculated at 2894.1 IU and 15084.4 pM/L for the normal group and at 2361.5 IU and 12484.7 pM/L for the abnormal group (Fig. 8D,E), which was not significantly different (p = 0.18 and 0.20, respectively). Excluding patients with more than one embryo, dose and estrogen response (E_2_) was 4150 IU and 15394.3 pM/L for the normal group and 2233.3 IU and 11546.6 pM/L for the abnormal group (results not shown), which was not significantly different (p = 0.06 and p = 0.43, respectively). For fertilization method, percentage of embryos in the normal group was 62.5% IVF and 47.5% ICSI, and in the abnormal group was 57.7% IVF and 42.3% ICSI (Fig. 8F), which did not differ statistically (p = 0.33). For day 3 embryo grade, embryos with normal methylation levels exhibited a grade of slight C/G2 (slC/G2) (3 embryos) and C/G3 (1 embryo) while those with abnormal methylation levels had a grade of A/B/G1 (4 embryos), slC/G2 (8 embryos) and C (1 embryo) (Fig. 8G). Importantly, embryos transferred to patients (Supplementary Table 3 online, 28 A/B/G1, 15 slC/G2 and 12 C/G3) had similar grading information to those that were frozen. For blastocysts, 10 of the 13 embryos with normal methylation levels had grading information; 3 were AA, 1 AB, 1 BA, 2 BB, 1 BC, 1CA and 1 CB (Fig. 8H). For embryos with abnormal methylation levels, 6 of the 13 had grading information: 5 were AA and 1 BA. These grades were not statistically different (p = 0.25). A comparison of these grading criteria separately showed that for stage (all 26 embryos included), embryos with normal methylation (5 stage 2, 2 stage 3, 6 stage 4) were not significantly different (p > 0.99) from embryos with abnormal methylation (1 stage 1, 7 stage 2, 3 stage 3, 2 stage 4). For ICM grade, embryos with normal methylation (4 A, 4 B, and 2 C) were not statistically different (p = 0.40) than embryos with abnormal methylation levels (5 A, 1 B). For TE grade, embryos with normal methylation (5 A, 4 B, 1 C) were not statistically different (p = 0.60) from embryos with abnormal methylation levels (6 A). Overall, no specific parameter was identified to have an association with abnormal imprinted methylation. Importantly, we found that embryos of the highest quality with day 3 A/B/G1 and blastocyst AA grading can have with abnormal methylation.

## Discussion

Although mouse models have been instrumental in analyzing the effects of ARTs on genomic imprinting in oocytes and early embryos, it is important to assess the effects of these technologies in donated human counterparts. This is especially important, as imprinting errors were perceived to be more common in mouse preimplantation embryos than in ART-conceived children. In this study, we observed that 76% day 3 embryos exhibited perturbed imprinted methylation, with 56%, 86% and 71% day 3 embryos possessing aberrant *SNRPN*, *KCNQ1OT1* and *H19* imprinted methylation, respectively. Furthermore, 50% blastocyst-stage embryos exhibited abnormal methylation levels with 67%, 31% and 14% blastocysts having aberrant *SNRPN*, *KCNQ1OT1* and *H19* imprinted methylation, respectively. Both losses and gains of imprinted methylation were observed, and in some case, both within the same embryo (ex. 17C1, 22B1). Additionally, 50% of day 3 and one blastocyst embryo exhibited both *KCNQ1OT1* and *H19* imprinted methylation perturbations (6C1, 7C1, 7C2, 14B2). This is similar to the multi-locus loss of imprinting we previously observed in the mouse^29^ and that others have reported in BWS and SRS children^4^,^15^,^21^,^22^,^27^,^36^,^37^,^38^,^39^.

Very few studies have examined the effects of ARTs on genomic imprinting in donated human preimplantation embryos^34^,^35^,^40^,^41^,^42^,^43^. Moreover, these studies were primarily performed on poor quality embryos that were unsuitable for transfer. Nevertheless, their results were similar to what is reported here. For *SNRPN*, 8/9 day 3 embryos (89%) possessed a loss or gain of methylation^42^. For *KCNQ1OT1*, 7/67 day 3 embryos (10%)^40^ and 9/16 poor quality blastocysts (56%) harboured aberrant methylation^34^. Finally for *H19*, 3 studies reported aberrant imprinted methylation in 6/32 day 3 embryos (17%)^41^, 9/21 poor quality morula-blastocysts (43%)^35^, and 5/60 blastocysts (8%)^40^, while the remaining study did not observe any alterations in *H19* imprinted methylation in 8 low quality blastocysts (0%)^34^. In addition to these genes, previous studies identified 11/65 day 3 embryos (17%) with abnormal *PEG1* imprinted methylation^40^ and 18/24 day 3 embryos (75%) with aberrant *GTL2* imprinted methylation^43^. All together, our study along with previous publications demonstrates that the frequency of imprinting errors in human donated preimplantation embryos (6–89%) occurs at a similar frequency to that produced in mouse preimplantation embryos (10–90%)^29^,^30^,^31^,^33^.

Of the above studies, two examined imprinted methylation in good quality, *in vitro* produced embryos. For *KCNQ1OT1*, 2/5 high quality (40%) blastocysts harboured aberrant methylation^34^, which was similar to what we report here (4/13; 31%). For *H19*, 0/5 high quality (0%) morula-blastocysts^35^ and 0/5 high quality blastocysts (0%) possessed aberrant methylation^34^. This contrasted with our study where we observed 2/14 blastocysts (14%) with aberrant *H19* methylation. This discrepancy may relate to the number of embryos analyzed in these studies.

The design of our study allowed comparison of short culture to day 3 cleavage stages and extended culture to the blastocyst stage. Our data together with previous studies found imprinted methylation errors at both stages; *SNRPN* day 3 (56%, 89%) versus blastocysts (67%); *KCNQ1OT1* day 3 (86%, 10%) versus blastocysts (31%, 40%, 56%); and *H19* day 3 (71%, 17%) versus blastocysts (14%, 8%, 0%)^34^,^35^,^40^,^41^. Thus, the presence of methylation errors in embryos undergoing both short (55% embryos) and extended (31% embryos) culture indicates that methylation errors arise as early as the 6–8 cell stage. Furthermore, extending culture from day 3 to the blastocyst stage does not appear to pose any greater risk for imprinting errors. Consequently, our study offers additional support for extended culture to the blastocyst stage to select the most developmentally competent embryos.

Although the frequency of imprinting errors was similar between mice and human preimplantation embryos, disparity still exists between frequencies of imprinting errors in human preimplantation embryos compared to frequencies of imprinting errors reported in ART children. One explanation for this discrepancy may be that imprinting errors in the early embryo lead to reduced levels of implantation or pregnancy failure. Alternatively, blastomeres with aberrant imprinted methylation may be preferentially relegated to the extraembryonic lineages. Previous studies in the mouse provide support for the latter explanation, since we and others have observed a selective loss of imprinting in the placenta compared to the embryo in midgestation mouse embryos following preimplantation development in culture^32^,^44^,^45^.

Infertility rates have increased around the world^1^,^46^. Advanced maternal age (*>*35 years) is directly related to this rise, consequently leading to the question of whether delayed childbearing in ART women may contribute to increased imprinting errors in ART children. Additionally, current evidence indicates that the supra-physiological hormonal milieu of ovarian stimulation may produce adverse outcomes in ART pregnancies. For example, similar incidences of low birth weight and preterm low birth weight were present in ART children produced from donor oocytes from fertile women compared to oocytes from women compared to oocytes from subfertile mothers^47^. This birth weight variation in *in vitro*-conceived children may be explained by alterations in DNA methylation levels at growth-related genes, as detected in newborn cord blood and placenta^48^. With respect to imprinting disorders, ovarian stimulation has also been linked to BWS and AS in ART-conceived children^7^,^11^,^38^, and for some of these children, the only procedure used was ovarian stimulation^11^,^38^. Our comparison of maternal age, induction method, hormone dosage levels and stimulation response in embryos with and without aberrant methylation revealed no significant difference between these groups. These results were not all that surprising, since embryos with and without methylation errors may have had similar exposures to maternal factor treatment and/or parental biometrics; and all embryos were generated using supra-physiological hormone dosages and the same conditions for *in vitro* culture and slow-freezing cryopreservation. Similarly, no significant difference in fertilization method (IVF/ICSI) or blastocyst grade was observed between embryos with normal or abnormal imprinted methylation. However, it should be noted that even the highest quality day 3 cleavage (A/B/G1) and blastocyst-stage (AA) embryos harbour abnormal methylation levels. This finding has significant bearing on future studies employing high quality embryos as their control group. One further observation of note was that two embryos (19B1, 20B1), produced via donor sperm for male factor infertility, possessed abnormal imprinted methylation. This suggests that imprinting errors in these embryos cannot be explained by inherent infertility, but instead may point to ART-induced errors. Further studies are required to investigate imprinted methylation errors in *in vitro*-produced embryos using donor oocytes and sperm.

There were several limitations of this study. Similar to other studies on ART human embryos, our investigation lacks naturally conceived controls, which is unavoidable. Additionally, due to limited availability of donated embryos, this study and others employed small numbers in analyses. However, the statistical analyses used in this type of study remains valid within the embryo population analyzed, and may allow cumulative analysis of larger sample sizes in the future. Finally, although our study controlled for operating procedure in the clinic, donated embryos analyzed here were obtained from a single fertility clinic.

Going forward, future research should focus on determining differences between human embryos with and without imprinting errors with respect to embryo properties, the timing and origin of these errors, as well as the molecular factors responsible for inducing imprinted methylation errors in ART embryos. Animal models will be instrumental in these studies prior to investigation in human embryos.

## Materials and Methods

### Donated human embryos

Twenty-three patients who had completed their fertility treatment at The Fertility Clinic at London Health Sciences Centre donated for research 24 day 3 cleavage and 29 blastocyst-stage human embryos that were no longer needed for their treatment. Buccal cells (B1-B4) were obtained from 4 healthy, non-patient adults (<30 years old). Research ethics approval was obtained through the Western University’s Health Science Research Ethics Board (102659) and the methods were carried out in accordance with the approved guidelines. Informed consent was obtained from patients donating embryos and non-patient adults providing buccal cell samples. All embryos were cultured in the glucose/phosphate-free preimplantation stage 1 (P1) culture medium (Irvine Scientific, California) to day 3, then in Blastocyst Medium (BM) in a sequential media protocol (Irvine Scientific, California) to the blastocyst stage. Embryos were slow frozen between the years 2000–2007 and thawed between October 2013-August 2014. Slow freezing was performed according to the Testart’s (propanediol) freezing method^49^ using Sydney IVF Cryopreservation Kits.

Day 3 human embryos were graded by blastomere number, and morphological fragmentation levels by either the former A through F grading system or the currently used G1 through G6 system: A, even, regular, no fragments; B, uneven, irregular, no fragments; slight C (slC), slight fragmentation; C, minor (<25%) fragmentation; D, major (between 25–50%) fragmentation; E, extensive (>50%) fragmentation; F, degenerate; or by fragmentation levels: G1, <5% fragmentation; G2, 5–10% fragmentation; G3, 11–25% fragmentation; G4, 26–50% fragmentation; G5, >50% fragmentation; and G6, degenerate^50^,^51^,^52^. Following thawing, the majority of embryos were G1-G3 grade and had an average of 4 cells (data not shown).

Blastocyst grading was according to blastocyst cavity size/hatching, inner cell mass characteristics and trophoblast characteristics giving a numeric-alpha-alpha score based on the Gardner and Schoolcraft scoring system^53^. Cavity size or hatching score was graded as 1, early blastocyst with cavity less than half the embryo volume; 2, blastocyst with cavity greater than half the embryo volume; 3, full blastocyst, cavity full; 4, expanded blastocyst, cavity expanded beyond earlier embryo size with thinning zona; 5, hatching blastocyst; 6, hatched blastocyst. Inner cell mass (ICM) grading was A, tightly packed ICM, many cells; B, loosely grouped ICM, several cells; and C, very few cells, and trophectoderm was graded as A, many cells with cohesive epithelium; B, few cells with loose epithelium; and C, very few large cells. All embryos were immediately processed for methylation analysis following thawing.

### Isolation of Control Cells

Buccal cells were collected using the end of a sterile 20 μL pipet tip and diluted into approximately 1000, 100, 50 and 5–10 cells in 20 μL of 1 X PBS (Phosphate-Buffered Saline). Buccal cells were then embedded into a 2:1 3% LMP agarose and lysis solution, and then subjected to imprinted DNA methylation analysis. One confluent well of a 6-well dish (~1 × 10^6^ cells) of HES2 human ESCs (WiCell Research Institute Inc.) was washed once with 1X PBS (Sigma) and incubated in TrypLE Express (GIBCO) in Dulbecco’s PBS (DPBS). Trypsin was inactivated by addition of DMEM and 10% Fetal Bovine Serum (FBS) medium. Detached hESCs were collected, pelleted gently, washed with 1X PBS and re-suspended in 1000 μL of 1X DPBS. Approximately 1 μL of cells (~1000 cells) was embedded into a 2:1 3% LMP agarose and lysis solution, then subjected to bisulfite mutagenesis.

### Imprinted DNA Methylation Analysis

Immediately following thawing individual embryos were embedded under mineral oil (Sigma) into 10 μL of a 2:1 mixture of 3% LMP agarose (Sigma) and lysis solution [100 mM Tris–HCl, pH 7.5 (Bioshop), 500 mM LiCl (Sigma), 10 mM EDTA, pH 8.0 (Sigma), 1% LiDS (Bioshop), and 5 mM DTT (Sigma), 1 μl 2 mg/ml proteinase K (Sigma), and1 μl 10% Igepal (Sigma)]. DNA methylation analysis was performed using the bisulfite mutagenesis and clonal sequencing method as previously described^54^. Samples were placed on ice for 10 minutes to generate an agarose/lysis bead and subsequently incubated overnight in SDS lysis buffer for 20 hours in a 50 °C water bath. Lysis buffer was removed and replaced with 300 μL of mineral oil and embryos were either frozen at −20 °C for a maximum of 3 days or immediately processed for bisulfite mutagenesis. Briefly, for bisulfite treatment, samples were incubated at 90 °C to inactivate proteinase K (Sigma) for 2.5 minutes and transferred to ice for 10 minutes. DNA denaturation was performed in 1 mL of 0.1 M NaOH at 37 °C for 15 minutes. Samples were covered with 300 μL of mineral oil and 500 μL of 2.5 M bisulfite solution for a 3.5-hour bisulfite conversion at 50 °C. After conversion, desulfonation was performed in 1 mL of 0.3 M NaOH at 37 °C for 15 minutes. Negative controls (beads containing no embryo or buccal cell sample) were processed with each bisulfite reaction. For first round PCR amplification, agarose bead with bisulfite converted DNA (10 μL) was added directly to 15 μL of Hot Start Ready-To-Go (RTG) (GE Healthcare) PCR bead that contained 0.5 μL of each 10 μM gene-specific external primer, 1 μL of 240 ng/mL transfer RNA and water with a 25 μL mineral oil overlay. Multiplexing of *H19* and *KCNQ1OT1* was performed during the first round of PCR. *SNRPN* amplification was performed individually. Five microliters of first round PCR product was added to 20 μL of RTG beads mixed with 19 μL 0.5 μL of each 10 μM internal primer and water for nested PCR. Separate second round PCR reactions were performed for *H19* and *KCNQ1OT1.*

*The KCNQ1OT1* PCR bisulfite primers were described previously^34^,^35^. The *KCNQ1OT1* region analyzed contained a G (94.7%)/A (6.3%) SNP (Rs56134313). For the *H19* region (GenBank Af087017, 6161-6409), external primers used were as described previously^34^. Due to SNPs residing in the previously described inner primers^34^, newly designed forward inner primer 5′-TTGGTTGTAGTTGTGGAAT-3′ and *H19* reverse inner primer 5′-AACCATAACACTAAAACCCT-3′ were used for nested PCR, amplifying a 249 base pair sequence encompassing 20 CpGs and Rs2071094 A (33.6%)/C (66.4%) and Rs2107425 G (55.5%)/A (44.5%) common SNPs. For *SNRPN*, nested primers (UCSC, chr15:25, 200, 009-25, 200, 379) were designed to amplify a 360 base pair region encompassing 24 CpGs and a G (84.8%)/A (15.2%) SNP (Rs220029) within the ICR as follows, *SNRPN* outer forward, 5′-TAGTGTTGTGGGGTTTTAGGG-3′; *SNRPN* outer reverse, 5′-TACCCACCTCCACCCATATC-3′; *SNRPN* inner forward, 5′-AGGGAGGGAGTTGGGATTT-3′; *SNRPN* inner reverse, 5′-CACAACAACAAACCTCTAAACATTC-3′. All PCR reactions were performed as previously described^55^, 94 °C for 10 minutes followed by 55 cycles of 94 °C for 15 seconds, 56 °C for 20 seconds and 72 °C for 20 seconds, with a final 72 °C for 10 minute extension.

PCR products were ligated into the pGEM-T EASY vector system (Promega), transformed into Z-competent DH5α *Escherichia coli* cells (Zymo Research) and following blue/white selection and colony PCR, samples were sent for sequencing at Bio Basic Inc. (Markham, ON, Canada)^29^. For both day 3 and blastocyst-stage embryos, 32–64 clones were sequenced per embryo per gene. Methylation patterns were determined using online software (BISMA). Identical clones (identical location and number of unconverted CpG-associated cytosines and identical location and number of unconverted non-CpG-associated cytosines) were included only once and represented one individual DNA strand. Only clones with ≥85% conversion rates were included. Total DNA methylation for each gene, or for each allele of a gene, if parental identity was assigned, was calculated as a percentage of the total number of methylated CpG/the total number of CpG dinucleotides.

### Statistical Analysis

Student’s t-test was used to examine significance between embryos with normal methylation and those with abnormal methylation for maternal age, hormone dose, and stimulation response (E_2_ levels). Statistical analyses for patient diagnosis, hormone induction method, fertilization method, and embryo grade was determined using the nonparametric Kolmogorov-Smirnov (KS) test to analyze differences between groups. A p-value of <0.05 was considered to be statistically different.

## Additional Information

**How to cite this article**: White, C. R. *et al.* High Frequency of Imprinted Methylation Errors in Human Preimplantation Embryos. *Sci. Rep.* **5**, 17311; doi: 10.1038/srep17311 (2015).

## Supplementary Material

Supplementary Information

Supplementary Table 2
