# Supplementary material for: High Frequency of Imprinted Methylation Errors in Human Preimplantation Embryos
Source: Sci Rep. 2015 Dec 2;5:17311. doi: 10.1038/srep17311 (PMC4667293; doi:10.1038/srep17311)
Supplement: Supplementary Information [file srep17311-s1.pdf]

Supplementary Information

High Frequency of Imprinted Methylation Errors in Human Preimplantation Embryos

Carlee R. White, Michelle M. Denomme, Francis R Tekpetey, Valter Feyles, Stephen GA Power, Mellissa R.W. Mann

Supplementary Table 1. Buccal cell sample, hESCs and embryo genotype

Study ID	*SNRPN* Rs220029 (Mat/Pat)*	*KCNQ1OT1* Rs56134313 (Mat/Pat)*	*H19* Rs2071094 (Mat/Pat)*	*H19* Rs2107425 (Mat/Pat)*
**Controls**				
Bu1	G/G	G/G	A/A	G/G
Bu2		G/G	T/T	A/A
Bu3	G/G	G/G	T/A	A/G
Bu4	G/G		A/T	G/G
**hESCs**	G/G	G/G		
**Day 3**				
1C1	G/G			
1C2	G/G			
1C3	G/G			
1C4	G/G			
1C5	G/G			
1C6	G/G			
9C2	G/G			
18C1	G/G			
21C1	G/G			
3C1		G/G	A/A	G/G
3C2		IC	IC	IC
4C1		G/G	T/A	A/G
6C1		G/G	T/T	A/G
6C2		IC	T/A	G/G
7C1		G/G	T/A	A/G
7C2		G/G	T/A	A/G
9C1		G/G	A/A	A/G
12C1		G/G	IC	
**Blastocyst**				
9B2	A/G			
10B1	G/G			
10B2	G/G			
10B3	G/G			
14B3	G/G			
14B4	G/G			
14B5	G/G			
16B1	G/G			
16B2	G/G			
17B1	A/G			
22B1	G/A			
23B1	G/G			
2B1		G/G	T/A	G/G
2B2		G/G	T/T	G/A

4B1	G/G	T/A	A/G
4B2	G/G	A/A	G/G
8B1	G/G	T/A	A/G
9B1	G/G	A/T	G/G
11B1	G/G	A/A	G/G
13B1	G/G	T/T	A/G
14B1	G/G	A/A	G/G
14B2	G/G	T/A	A/G
15B1	G/G	A/A	G/G
19B1	G/G	A/T	G/G
20B1	G/G	A/A	G/G
21B1	IC	A/A	G/G

* presumptive maternal and paternal alleles

Bu, buccal cell samples; hESCs, human embryonic stem cells; Mat, maternal; Pat, paternal; C, day 3 cleavage stage embryo; B, blastocyst stage embryo; ND, not determined; IC, inconclusive.

Supplementary Table 3. Pregnancy outcome for each patient

Patient	# ET	Embryo grade	Pregnancy	Live birth	BW (g)	Category
1	2	8B,10slC	No	-		
2	3	8B,8B,7B	No	-		
3	2	9slC,8A	Yes	Y (twins)	925, 820	EL,EL
4	2	8B, 9B	No	-		
5	3	8A,8A,8slC	Yes	Y (twins)	2240, 2466	L,L
6	3	10slC,8A,8A	Yes	Y	1185	VL
7	2	9slC,10slC	Yes	Y	3856	N
8	2	10C,8B	No	-		
9	3	8B,8slC,8A	Yes	Y (twins)	2722, 2665	N,N
10	2	8slC,9C	Yes	Y	3600	N
11	2	8slC,6slC	Yes	Y	3260	N
12	3	8A,7C,A*	Yes	Y	2920	
13	3	7C,4B,5C	No	-		
14	3	8A,8B,8B	Yes	Y (triplets)	2268, 2551, 2551	L, N, N
15	2	7C,10C	No	-		
16	2	6slC,6C	Yes	N		
17	2	7B,7slC	Yes	Y	2948	N
18	2	8B,10B	Yes	Y	4678	H
19	2	8slC,10C	No	-		
20	3	8B,8slC,8A	Yes	Y	3912	N
21	3	8C,8C,8C	Yes	Y (twins)	2325, 2041	L,L
22	2	7A,9A	Yes	Y	3515	N
23	2	10slC,8A	No	-		

ET, embryos transferred; BW, birth weight; N, normal BW; L, low BW; VL, very low BW; EL, extremely low BW. Asterisk indicates embryo was compacting.

Supplementary Table 4. Patient biometrics and clinical treatment

Emb ID	Mat Age	Diagnosis	Induction Method	Dose (IU)	E_2 Levels (pM/L)	IVF/ICSI	Freeze Year	Emb Grade
1C1	34	MF	Agonist, Synarel, Gonal-F	1,800	16,287	ICSI	2005	3C
1C2	34	MF	Agonist, Synarel, Gonal-F	1,800	16,287	ICSI	2005	7C
1C3	34	MF	Agonist, Synarel, Gonal-F	1,800	16,287	ICSI	2005	6slC
1C4	34	MF	Agonist, Synarel, Gonal-F	1,800	16,287	ICSI	2005	7slC
1C5	34	MF	Agonist, Synarel, Gonal-F	1,800	16,287	ICSI	2005	4slC
1C6	34	MF	Agonist, Synarel, Gonal-F	1,800	16,287	ICSI	2005	7A
3C1	30	MF	Agonist, Synarel, Gonal-F	1,650	16,305	ICSI	2006	7G2
4C1	32	BTO	Agonist, Synarel, Gonal-F	2,250	22,206	IVF	2005	6B
6C1	35	IDIO+MF	Agonist, Synarel, Gonal-F	1,950	8,934	ICSI	2006	7A
6C2	35	IDIO+MF	Agonist, Synarel, Gonal-F	1,950	8,934	ICSI	2006	7slC
7C1	26	PCOS	Antagonist, Orgalutran, Puregon	1,100	6,155	IVF	2007	8slC
7C2	26	PCOS	Antagonist, Orgalutran, Puregon	1,100	6,155	IVF	2007	7slC
9C1	36	BTO+ENDO	Antagonist, Orgalutran, Bravelle	4,200	7,446	ICSI	2006	8B
9C2	36	BTO+ENDO	Antagonist, Orgalutran, Bravelle	4,200	7,446	ICSI	2006	8slC
12C1	37	BTO+ENDO	Agonist, Synarel, Gonal-F	4,275	4,086	IVF	2007	8G2
18C1	32	BTO	Agonist, Synarel, Gonal-F	1,650	5,565	IVF	2004	8slC
21C1	42	AMA	Agonist, Lupron, Repronex	2,850	29,613	IVF	2006	7slC
2B1	38	BTO+MF	Antagonist, Orgalutran, Gonal-F	5,850	10,416	ICSI	2004	4BB
2B2	38	BTO+MF	Antagonist, Orgalutran, Gonal-F	5,850	10,416	ICSI	2004	2AA
4B1	32	BTO	Agonist, Synarel, Gonal-F	2,250	22,206	IVF	2005	3BC
4B2	32	BTO	Agonist, Synarel, Gonal-F	2,250	22,206	IVF	2005	4CA
8B1	34	TD	Agonist, Synarel, Gonal-F	1,800	18,588	IVF	2003	2--
9B1	36	BTO+ENDO	Antagonist, Orgalutran, Bravelle	4,200	7,446	ICSI	2006	1AA
9B2	36	BTO+ENDO	Antagonist, Orgalutran, Bravelle	4,200	7,446	ICSI	2006	3AA
10B1	32	PCOS	Agonist, Synarel, Gonal-F	1,350	9,118	IVF	2006	4AB
10B2	32	PCOS	Agonist, Synarel, Gonal-F	1,350	9,118	IVF	2006	4BB
10B3	32	PCOS	Agonist, Synarel, Gonal-F	1,350	9,118	IVF	2006	4BA
11B1	35	MF+FPES	Agonist, Synarel, Gonal-F	2,250	3,345	ICSI	2007	4AA
13B1	36	IDIO	Antagonist, Orgalutran, Menopur	6,300	14,882	ICSI/IVF	2007	4BA
14B1	36	BTO	Agonist, Synarel, Gonal-F	2,700	15,373	IVF	2007	4AA
14B2	36	BTO	Agonist, Synarel, Gonal-F	2,700	15,373	IVF	2007	3AA
14B3	36	BTO	Agonist, Synarel, Gonal-F	2,700	15,373	IVF	2007	2--
14B4	36	BTO	Agonist, Synarel, Gonal-F	2,700	15,373	IVF	2007	2--
14B5	36	BTO	Agonist, Synarel, Gonal-F	2,700	15,373	IVF	2007	2--
15B1	30	ANOV	Agonist, Synarel, Gonal-F	4,350	12,713	IVF	2003	2--
16B1	31	BTO	Agonist, Synarel, Gonal-F	1,800	13,057	IVF	2007	2AA
16B2	31	BTO	Agonist, Synarel, Gonal-F	1,800	13,057	IVF	2007	2AA
17B1	35	BTO+ENDO	Agonist, Synarel, Fertinorm	1,950	7,555	IVF	2000	2--
19B1	32	MF (DONOR)	Puregon, no agonist	1,300	13,158	IVF	2002	4--
20B1	33	MF (DONOR)	Agonist, Synarel, Gonal-F	3,375	10,502	ICSI	2003	2--
21B1	42	AMA	Agonist, Lupron, Repronex	2,850	29,613	IVF	2006	2CB
22B1	23	BTO+PCOS	Agonist, Synarel, Gonal-F	2,475	13,809	IVF	2003	4--
23B1	26	BTO	Agonist, Synarel, Gonal-F	1,275	28,132	IVF	2003	2--

Emb, embryo; Mat, maternal; C, day 3 cleavage stage embryo; B, blastocysts; E_2, serum estrogen on day of hCG trigger; ICSI, intracytoplasmic sperm injection; IVF, *in vitro* fertilization; MF, male factor; ENDO, endometriosis; BTO, bilateral tubal obstruction/occlusion; IDIO, idiopathic; PCOS, polycystic ovarian syndrome; AMA, advanced maternal age; ANOV, anovulatory; TD, tubal disease; DONOR, donor sperm; FPES, Fresh/frozen percutaneous epididymal/testicular sperm aspiration sample; --, no grade available.

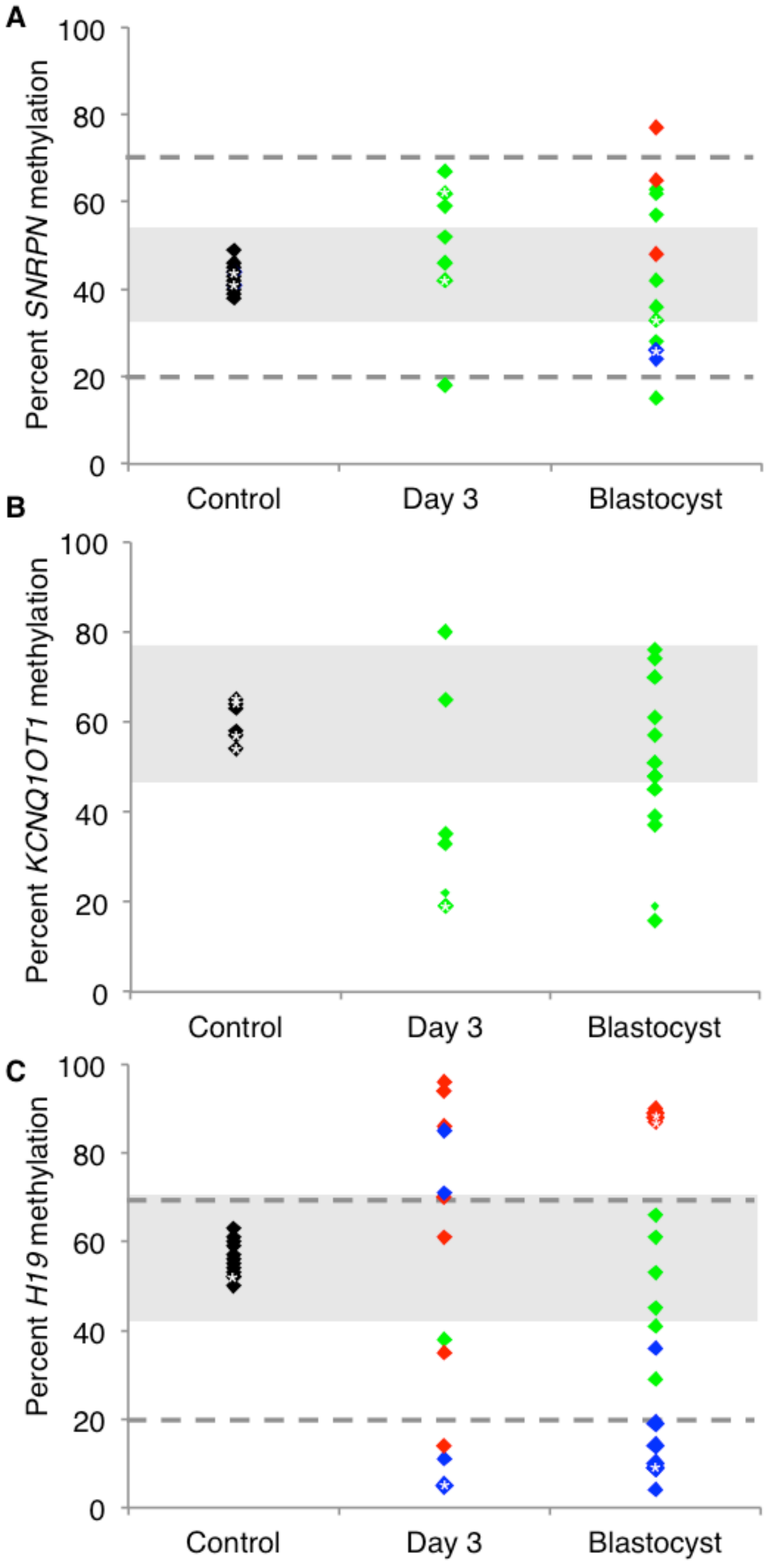

Supplementary Figure 1. Graphical representation for (A) *SNRPN*, (B) *KCNQ1OT1* and (C) *H19* methylation levels in control buccal and ESC samples, and day 3 cleavage and blastocyst-stage embryos. Black diamonds, control sample methylation levels with grey shaded area indicating normal methylation range. Green diamonds, total methylation levels in day 3 embryos and blastocyst embryos. Red diamonds indicate presumptive *SNRPN*, *KCNQ1OT1* and *H19* maternal alleles and blue diamonds indicate presumptive *SNRPN*, *KCNQ1OT1* and *H19* paternal alleles, with grey dashed lines representing ≥70% methylation and ≤20% methylation allelic cutoffs. Asterisk (*) represents a data point for which more than one embryo exists.
